# The Herbal Medicine KIOM-MA128 Inhibits the Antigen/IgE-Mediated Allergic Response in Vitro and in Vivo

**DOI:** 10.3390/molecules21081015

**Published:** 2016-08-04

**Authors:** Kwang Il Park, Dong Gun Kim, Jae Myung Yoo, Jin Yeul Ma

**Affiliations:** Korean Medicine (KM)-Application Center, Korea Institute of Oriental Medicine (KIOM), 70 Cheomdan-ro, Dong-gu, Daegu 41062, Korea; kipark@kiom.re.kr (K.I.P.); kite79@kiom.re.kr (D.G.K.); jmyoo@kiom.re.kr (J.M.Y.)

**Keywords:** mast cell, allergic response, passive cutaneous anaphylaxis (PCA), FcεRI signaling, KIOM-MA128

## Abstract

KIOM-MA128, a novel herbal medicine, has been reported to exert some beneficial effects on various biological events, such as atopic dermatitis, inflammation and cancer. The aim of this study is to investigate how KIOM-MA128 regulates the allergic response. We measured the activity of β-hexosaminidase and the levels of allergic mediators in the conditioned media of antigen/IgE (Ag/IgE)-activated RBL-2H3 mast cells. We examined the levels of proteins associated with both the FcεRI and arachidonate cascades. Finally, we established the passive cutaneous anaphylaxis (PCA) model in mice to confirm the anti-allergic effects of KIOM-MA128 in vivo. KIOM-MA128 dose-dependently inhibited degranulation and the production of the allergic mediators described above, with no significant cytotoxicity. In the arachidonate cascade, KIOM-MA128 significantly reduced both cytosolic phospholipase A_2_ (cPLA_2_) phosphorylation and cyclooxygenase-2 (COX-2) expression. Moreover, in the FcεRI cascade, KIOM-MA128 not only inhibited activation of LYN, FYN and SYK, known as the rate-limiting proteins of the FcεRI cascade, but also suppressed the phosphorylation of ERK, p38 and JNK, which is related to cytokine expression. Finally, 50 to 100 mg/kg KIOM-MA128 significantly attenuated the Ag/IgE-induced PCA reaction in mice. These findings provide novel information and improve our understanding of the anti-allergic effects of KIOM-MA128 on allergic diseases.

## 1. Introduction

Mast cells play a critical role in the allergic response, including early- and late-phase reactions [1]. A high-affinity receptor for IgE (FcεRI), which is located on the mast cell membrane, interacts with antigen-specific IgE (Ag/IgE). The interaction activates the mast cells by initiating molecular signaling pathways, such as the efflux of Ca^2+^ into the cytosol, and/or phosphorylation of tyrosine kinases, including SYK, LYN and FYN. Cross-linking FcεRI and the Ag/IgE complex activates LYN, which then activates SYK. These processes cause the activation of a signaling cascade, including LAT, phospholipase C (PLC) γ, and MAP kinases (MAPKs). Additionally, activation of mast cells by the Ag/IgE complex is associated with the secretion of granules containing various allergic mediators, such as β-hexosaminidase, histamines, eicosanoids, serotonin, and pro-inflammatory cytokines and chemokines. In particular, tumor necrosis factor-α, a pro-inflammatory cytokine, induces inflammation during degranulation and recruits various immune cells, causing severe inflammation [2]. In addition, the immortalized RBL-2H3 cell line is originated from rat basophilic leukemia cells, and is mostly used for the evaluation of mast cell degranulation and for screening potential anti-allergic agents [3,4].

*Glycyrrhizae radix*, *Polygoni cuspidati Rhizoma*, *Sophorae radix*, *Cnidii rhizoma* and *Arctii fructus* have been used in traditional oriental medicine in several Asian countries, including China and Korea. We formulated a new herbal medicine formula called KIOM-MA using these herbs. Our previous studies showed that KIOM-MA possessed anti-inflammatory properties in RAW 264.7 macrophages [5] as well as effects against atopic dermatitis (AD) [6]. Furthermore, our group fermented the KIOM-MA using probiotics to increase the absorption and bioavailability of the active ingredients [7], and named it KIOM-MA128. KIOM-MA128 has greater anti-cancer [8] and anti-inflammatory effects [5] than KIOM-MA. However, the cellular signaling mechanisms related to its anti-allergic actions are not yet known.

In the present study, we hypothesized that KIOM-MA128 might prevent allergic reactions in Ag/IgE-activated mast cells. We investigated the degranulation of Ag/IgE-stimulated RBL-2H3 cells by measuring β-hexosaminidase activity to examine the anti-allergic effects of KIOM-MA128. The levels of inflammatory mediators, such as tumor necrosis factor-α (TNF-α), histamine, interleukin-4 (IL-4), IL-6 and prostaglandin D_2_ (PGD_2_), were analyzed using enzyme immunoassay (EIA) and enzyme-linked immunosorbent assay (ELISA) kits to evaluate the anti-allergic effects of KIOM-MA128. The FcεRI signaling pathway was investigated by immunoblot analysis to confirm the anti-allergic mechanisms of KIOM-MA128. Finally, we performed Ag/IgE-mediated passive cutaneous anaphylaxis (PCA) reaction in mice to demonstrate the anti-allergic action of KIOM-MA128 in in vivo system. Here, we report that KIOM-MA128 suppresses Ag/IgE-induced allergic responses in RBL-2H3 cells. Furthermore, our findings support the clue to understanding the anti-allergic action of KIOM-MA128 in allergic diseases.

## 2. Results

### 2.1. KIOM-MA128 Did Not Affect Cell Viability in RBL-2H3 Mast Cells

**Figure 1:**
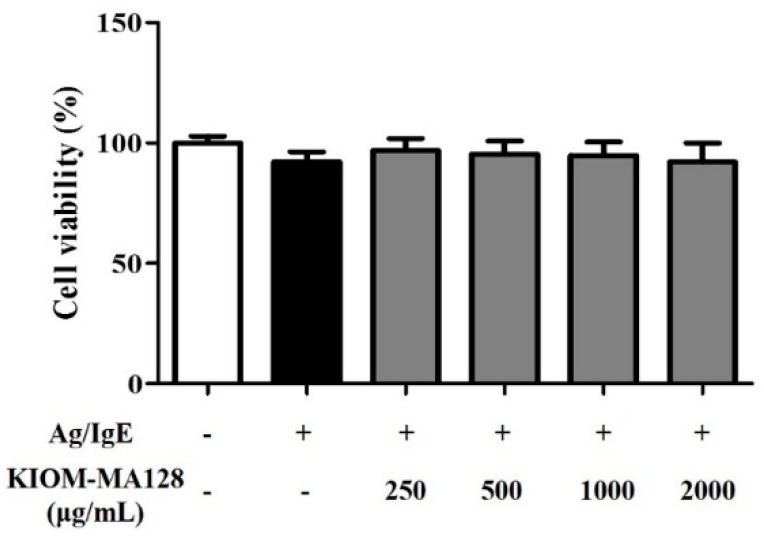
Effect of KIOM-MA128 on cell viability in IgE/Ag-activated RBL-2H3 mast cells. RBL-2H3 mast cells were seeded on a 96-well plate (1 × 10^4^ cells/well) in MEM-α with 10% FBS and incubated overnight at 37 °C. The cells were further incubated with DNP-IgE (0.1 μg) for 24 h and then treated with KIOM-MA128 (0–2000 μg/mL). After 1 h, they were stimulated with DNP-Ag (0.1 μg/mL) for 4 h. Cell viability was determined using the procedure described in the Materials and Methods section. The data represent the mean ± SD values of three independent experiments.

We measured the effects of KIOM-MA128 on the viability of RBL-2H3 cells. RBL-2H3 cell viability was measured following treatment with various concentrations of KIOM-MA128 (250–2000 µg/mL) for 24 h. The findings indicated that KIOM-MA128 did produce cytotoxicity in RBL-2H3 cells. The results indicated that KIOM-MA128 has not significant cytotoxicity (Figure 1).

### 2.2. KIOM-MA128 Inhibits Ag/IgE-Mediated Degranulation in RBL-2H3 Cells

**Figure 2:**
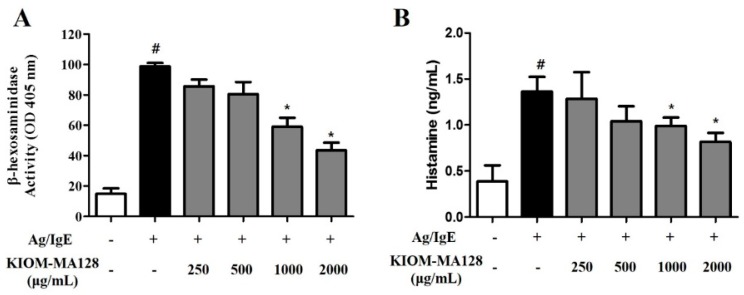
Inhibitory effects of KIOM-MA128 on degranulation and histamine release. IgE-sensitized RBL-2H3 mast cells were treated with KIOM-MA128 for 1 h before antigen challenge. β-hexosaminidase activity and the histamine concentrations were determined using the procedure described in the Materials and Methods section. The data represent the mean ± SD values of three independent experiments. # *p* < 0.05 versus the control group; * *p* < 0.05 versus the DNP-Ag-treated group. (**A**) β-hexosaminidase; (**B**) histamine.

We measured both β-hexosaminidase activity and the histamine concentrations in media from IgE-sensitized mast cells that were stimulated with antigen (0.1 µg/mL DNP-HSA) and various concentrations of KIOM-MA128 to investigate the modulatory effects of KIOM-MA128 on Ag/IgE-mediated degranulation and histamine release in RBL-2H3 cells. KIOM-MA128 significantly inhibited degranulation in Ag/IgE-induced RBL-2H3 cells in a dose-dependent manner (Figure 2A). Moreover, histamine release was markedly reduced by 1000 and 2.000 µg/mL KIOM-MA128 in Ag/IgE-activated RBL-2H3 cells (Figure 2B). These results showed that KIOM-MA128 ameliorated the allergic effects of the Ag/IgE reaction.

### 2.3. KIOM-MA128 Inhibits the IgE-Induced Release of Pro-Inflammatory Cytokines in RBL-2H3 Cells

**Figure 3:**
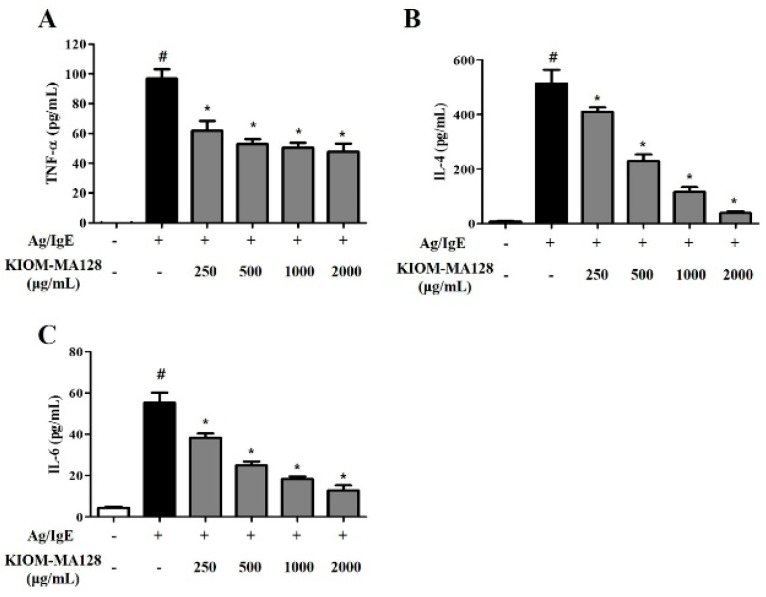
Inhibitory effects of KIOM-MA128 on the production of allergic mediators. IgE-sensitized RBL-2H3 mast cells were treated with KIOM-MA128 for 1 h prior to antigen treatment. The levels of TNF-α, IL-4 and IL-6 were determined using the procedure described in the Materials and Methods section. The data represent the mean ± SD values of three independent experiments. # *p* < 0.05 versus the control group; * *p* < 0.05 versus the DNP-Ag-treated group. (**A**) TNF-α; (**B**) IL-4; (**C**) IL-6.

We measured the levels of cytokines such as TNF-α, IL-6 and IL-4 in IgE-sensitized RBL-2H3 cells using ELISA to determine the effects of KIOM-MA128 on Ag/IgE-induced pro-inflammatory cytokine production in RBL-2H3 cells. Activated mast cells secreted various cytokines, which play critical roles in allergic responses, such as the chemoattractant response that leads to the recruitment of other immune cells and the regulation of severe allergic reaction [9,10]. When IgE-sensitized mast cells are activated by antigen, KIOM-MA128 significantly reduced the TNF-α, IL-6 and IL-4 levels (Figure 3). These results showed that KIOM-MA128 inhibited allergic reactions by preventing the release of pro-inflammatory cytokines from Ag/IgE-activated RBL-2H3 cells.

### 2.4. KIOM-MA128 Inhibits the Arachidonate Signaling Pathway in RBL-2H3 Cells

**Figure 4:**
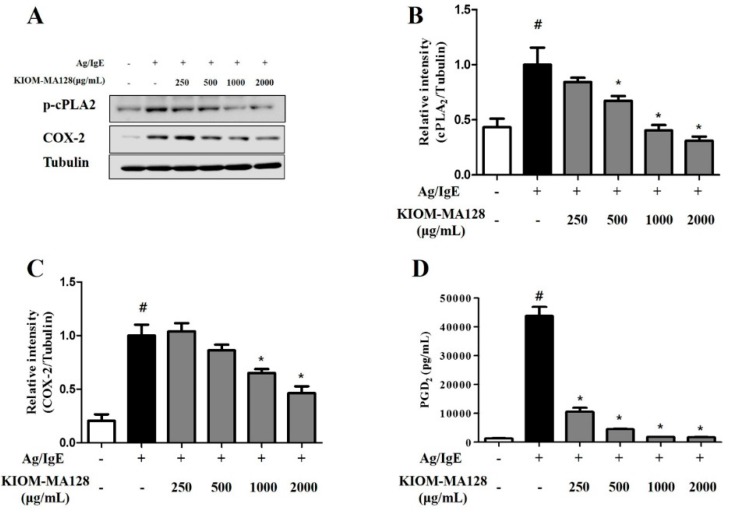
Inhibitory effects of KIOM-MA128 on PGD_2_ production and the activation of the arachidonate cascade. RBL-2H3 mast cells were seeded on a 6-well plate (5 × 10^5^ cells/well) in MEM-α with 10% FBS and incubated overnight at 37 °C. The cells were further incubated with DNP-IgE (0.1 μg) for 24 h and then treated with KIOM-MA128 (0–2000 μg/mL). After 1 h, they were stimulated with DNP-Ag (0.1 μg/mL) for 4 h. The amounts of PGD_2_ were determined as described in the Materials and Methods section. The data represent the mean ± SD values of three independent experiments. The cells were washed with 1× DPBS and lysed with cell lysis buffer. The levels of p-cPLA_2_, COX-2 and α-tubulin were determined using the procedure described in the Materials and Methods section. # *p* < 0.05 versus the control group; * *p* < 0.05 versus the DNP-Ag-treated group. (**A**) Immunoblot of p-cPLA_2_ and COX-2; (**B**) densitometry of p-cPLA_2_; (**C**) densitometry of COX-2; (**D**) PGD_2_.

We measured the PGD_2_, p-cPLA_2_ and COX-2 levels in Ag/IgE-activated RBL-2H3 cells using immunoblot analysis and ELISA kits to determine the effects of KIOM-MA128 on the levels of pro-inflammatory mediators in Ag/IgE-induced RBL-2H3 cells. cPLA_2_ is a rate-limiting enzyme in eicosanoid synthesis, and COX-2 converts arachidonic acid to prostaglandin. Therefore, cPLA_2_ and COX-2 are very important enzymes in the allergic response [11]. When IgE-sensitized mast cells are activated by antigen, KIOM-MA128 significantly decreased the cPLA_2_, COX-2 and PGD_2_ levels in dose-dependent manner (Figure 4). These results indicated that KIOM-MA128 suppressed the allergic reaction by inhibiting the arachidonate signaling pathway in RBL-2H3 cells.

### 2.5. KIOM-MA128 Inhibits the FcεRI Signaling Pathway in Ag/IgE-Activated RBL-2H3 Cells

**Figure 5:**
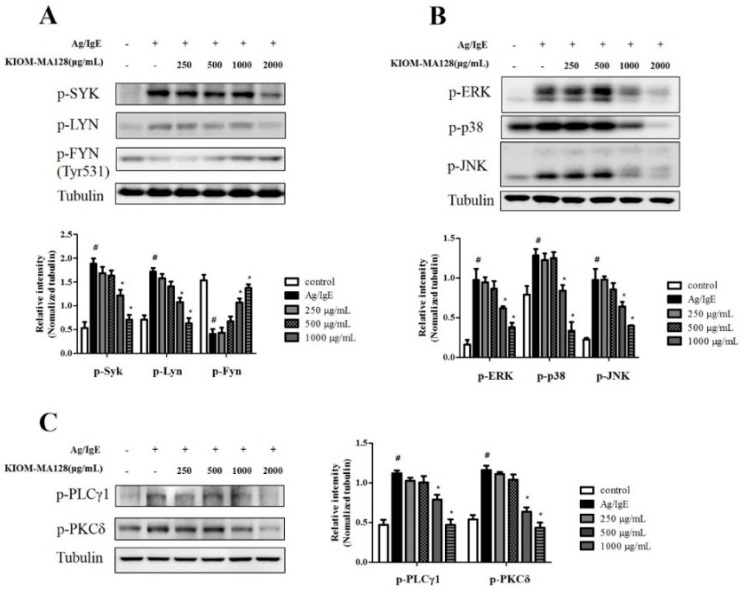
Inhibitory effects of KIOM-MA128 on the activation of the FcεRI cascade. IgE-sensitized RBL-2H3 cells were treated with KIOM-MA128 for 1 h and then stimulated with DNP-Ag for 10 min. The cells were washed with 1 × DPBS and lysed with cell lysis buffer. The levels of p-SYK, p-LYN, p-FYN, p-ERK, p-p38, p-JNK, p-PLCγ1, p-PKCδ and α-tubulin were determined using the procedure described in the Materials and Methods section. The data represent the mean ± SD values of three independent experiments. (**A**) p-SYK, p-LYN and p-FYN; (**B**) p-ERK, p-p38 and p-JNK; (**C**) p-PLCγ1 and p-PKCδ. # *p* < 0.05 versus the control group; * *p* < 0.05 versus the DNP-Ag-treated group.

We hypothesized that the suppression of the arachidonate signaling pathway by KIOM-MA128 resulted in the inhibition of the FcεRI signaling pathway in the early phase (10 min). We analyzed the expression of FcεRI cascade-related proteins to investigate the effects of KIOM-MA128 on the IgE-mediated FcεRI cascade. KIOM-MA128 reduced the levels of p-SYK and p-LYN, whereas it increased the phosphorylation levels of FYN (Tyr531), which is an inactive form [12]. Furthermore, KIOM-MA128 markedly reduced the expression levels of MAPKs, such as p-ERK, p-p38 and p-JNK. In addition, KIOM-MA128 significantly reduced the levels of p-PLCγ1 and p-PKCδ, major proteins involved in the degranulation of activated mast cells, in Ag/IgE-activated RBL-2H3 cells (Figure 5). These results indicated that KIOM-MA128 reduced the allergic reaction by inhibiting the FcεRI signaling pathway in RBL-2H3 cells.

### 2.6. KIOM-MA128 Inhibits the Allergic Response in the PCA Model

We used the passive cutaneous anaphylaxis (PCA) animal model to confirm that KIOM-MA128 exhibited anti-allergic effects in vivo. The ears of ICR mice were locally injected with IgE and then administered KIOM-MA128 for 1 h. A mixture of the antigens and Evans blue was then injected into the tail vein. 

**Figure 6:**
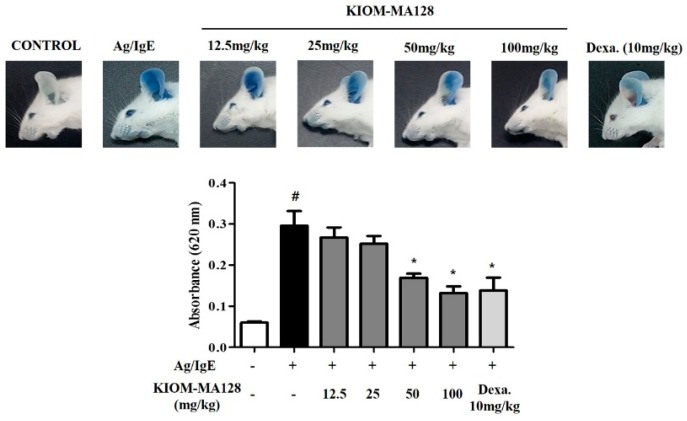
Inhibitory effect of KIOM-MA128 on Ag/IgE-induced passive cutaneous anaphylaxis in mice. IgE-sensitized mice were orally administered KIOM-MA128 (0–100 mg/kg) for 1 h and then intravenously injected with 100 μg DNP-HSA containing 0.5% Evans blue. After 30 min, the mice were euthanized, and then both ears were excised. The extravasated dye in the ears was analyzed using the procedure described in the Materials and Methods section. The data are listed as the mean ± SEM values from eight determinations. # *p* < 0.05 versus the control group; * *p* < 0.05 versus the DNP-Ag-treated group.

At 50 mg/kg and 100 mg/kg, KIOM-MA128 significantly inhibited the mast cell-mediated PCA reaction in mice, and it seemed like that the anti-allergic actions were better than dexamethasone (Figure 6).

## 3. Discussion

Previously, we reported that KIOM-MA possessed beneficial effects against allergic diseases, such as inflammation [5], atopic dermatitis [6]. In particular, its fermented form, KIOM-MA128, is known to have greater anti-inflammation efficiency than KIOM-MA [6,13]. In addition, we recently found that KIOM-MA128 had anti-cancer effects on melanoma and fibrosarcoma [8,14]. Nonetheless, it is unclear how KIOM-MA128 regulates the allergic responses in asthma and atopic dermatitis.

In this study, we show that KIOM-MA128 exerts anti-allergic actions in both Ag/IgE-activated RBL-2H3 cells and Ag/IgE-mediated PCA reaction in mice. These anti-allergic actions of KIOM-MA128 result from its ability to suppress both the degranulation process and the production of allergic mediators, such as histamine, IL-4, IL-6, TNF-α, and PGD_2_, in Ag/IgE-activated mast cells, and Ag/IgE-induced PCA reaction in mice. Moreover, KIOM-MA128 also regulates the activation of the FcεRI and arachidonate cascades in these cells. Therefore, the inhibitory effects of KIOM-MA128 may be closely associated with its ability to reduce the activation of Ag/IgE-stimulated mast cells. In support of this hypothesis, some components of KIOM-MA and KIOM-MA128, such as *Polygonum cuspidatum* [15], *Sophora flavescents* [16]*,* and *Arctium lappa* fruit [17], are known to possess anti-allergic properties in mast cells. Additionally, the fermentation using microorganisms is frequently used for ameliorating the beneficial effects of medicinal herbs [18,19]. All the take together, the results suggest that the anti-allergic efficiency of KIOM-MA128 in Ag/IgE-activated mast cells may result from the combination of the anti-allergic components of KIOM-MA128 and the fermentation process.

One possible mechanism for the anti-allergic actions of KIOM-MA128 in Ag/IgE-activated mast cells may be the direct suppression of the activation of the FcεRI cascade, because mast cells express FcεRI receptors on the extracellular face of the plasma membrane [20]. Actually, when IgE-sensitized mast cells are stimulated with antigens, the FcεRI cascade is activated by the immunoreceptor tyrosine-based activation motifs (ITAMs) through the clustering of this receptor [21]. Besides, the activated ITAMs trigger activation and recruitment of the rate-limiting proteins of the FcεRI cascade such as LYN and FYN, and then SYK is activated by LYN or/and FYN [22]. Activated SYK leads to elevation of intracellular Ca^2+^ levels, activation of MAPKs, which is associated with cytokine expression [23], and that of PLCγ1/2-PKCδ pathway, which is related with degranulation process [24]. As a result, Ag/IgE-activated mast cells liberate numerous granules containing various allergic mediators, such as histamine, inflammatory cytokines and prostaglandins, in a matter of minutes [25,26]. In support of this finding, when IgE-sensitized mast cells were stimulated with antigen, β-hexosaminidase activity known as a biomarker of degranulation, histamine, IL-4, IL-6, TNF-α and PGD_2_ levels were increased in the conditioned media of the cells. In contrast, KIOM-MA128 decreased these responses as well as the activation of LYN, FYN and SYK. Consequently, the phosphorylation of MAPKs including ERK, p38 and JNK, PLCγ1 and PKCδ is also reduced by KIOM-MA128. Therefore, an important role of KIOM-MA128 may be to regulate the activation of LYN, FYN or/and SYK to exert its anti-allergic properties.

Another possible mechanism may be associated with the inhibition of the arachidonate cascade in Ag/IgE-activated mast cells. Ag/IgE-activated mast cells can produce and liberate inflammatory prostaglandins and leukotrienes [27]. In particular, PGD_2_ is able to induce bronchoconstriction, increase in capillary permeability, mucous production, and vasodilation in asthma [28,29]. Besides, LTC_4_ as a strong spasmogenic and chemotactic biochemical can enhance the permeability of small vessels including capillaries, and play both promotion and maintenance of allergic inflammation by activating cysteinyl-LT receptors in allergic diseases such as asthma and allergic rhinitis [28,29]. Overall, PGD_2_ and LTC_4_ can sustain, and induce severe inflammation in allergic diseases. Therefore, the regulation of eicosanoid production is another important role of KIOM-MA128 in Ag/IgE-activated mast cells. In fact, in our data, KIOM-MA128 not only reduced the production of PGD_2_ but also decreased cPLA_2_ phosphorylation, which is a rate-limiting enzyme in the arachidonate cascade [30], and COX-2 expression, which is the rate-limiting enzyme for prostaglandin biosynthesis [31]. All the take together, cPLA_2_ and COX-2 may be targets of KIOM-MA128 and may mediate its anti-allergic properties.

## 4. Materials and Methods

### 4.1. Reagents

MEM-α medium, 1 × DPBS, penicillin, streptomycin and fetal bovine serum (FBS) were purchased from GE Healthcare Life Sciences (Hyclone™, Logan, UT, USA). The EZ-Cytox cell viability assay kit was obtained from Daeillab Service Co. (Seoul, Korea). Specific antibodies against phospho-cPLA_2_, phospho-ERK, phospho-JNK, phospho-LYN, phospho-p38, phospho-PKCδ, phospho-PLCγ1, phospho-SYK and COX-2 were purchased from Cell Signaling Technology, Inc. (Beverly, MA, USA). A specific antibody against phospho-FYN was obtained from Biorbyt Ltd. (Cambridge, UK). A specific antibody against α-tubulin was purchased from Santa Cruz Biotechnology, Inc. (Dallas, TX, USA). An enzyme immunoassay (EIA) kit for PGD_2_ was obtained from Cayman Chemical Co. (Ann Arbor, MI, USA). Enzyme-linked immunosorbent assay (ELISA) kits for TNF-α, IL-4, and IL-6 were purchased from e-Bioscience, Inc. (San Diego, CA, USA). 4-Nitrophenyl-*N*-acetyl-β-d-glucosaminide (p-NAG), dinitrophenyl-human serum albumin (DNP-HSA) and DNP-immunoglobulin E (DNP-IgE) were obtained from Sigma-Aldrich Co. (St. Louis, MO, USA). All other chemicals were of analytical grade.

### 4.2. Preparation of KIOM-MA128

KIOM-MA and KIOM-MA128 were prepared according to previously described methods [6]; the herbal medicines in KIOM-MA (*Glycyrrhizae radix*, *Polygoni cuspidati Rhizoma*, *Sophorae radix*, *Cnidii rhizoma*, *Arctii fructus*, etc. [6,14], was obtained from the Yeongcheon Oriental Herbal Market (Yeongcheon, Korea), and then identified by Dr. Ki-Hwan Bae, Professor Emeritus at the College of Pharmacy, Chungnam National University (Daejeon, Korea). KIOM-MA (1 kg) was boiled in distilled water (10 liters) for approximately 3 h at 115 °C. The aqueous extract was filtered through a testing sieve (Aperture 500 μm and 150 μm). The filtered extract was inoculated with *Lactobacillus rhamosus* (1 × 10^5^–10^7^ CFU/mL; KFRI 128, KCTC 2182) provided by the Korea Food Research Institute, the pH of which was adjusted to 7.0 with 1 N NaOH and then autoclaved for 5 min, and then incubated for 48 h at 37 °C. KIOM-MA128 was filtered through a nylon net filter (60 μm; Millipore Co., Denver, MA, USA), and then deposited overnight. The supernatant was lyophilized, and then the dried pellet (the yield, 20.44%) was stored at −20°C until use. KIOM-MA128 were dissolved in a 10% DMSO solution or deionized water for in vitro or in vivo studies, respectively.

### 4.3. Animals

ICR mice, known as Swiss CD-1 mice [32] (5 weeks, 25–30 g), were procured from Samtako (Osan, Korea) and housed in cages (5 mice per cage) under specific pathogen-free conditions (21–24 °C and 40%–60% relative humidity) with a 12 h light/dark cycle and were given free access to standard rodent food (Orientbio Inc., Sungnam, Korea) and water. All animal experiments were approved by the Animal Care and Use Committee of the KIOM (Daejeon, Korea) with reference number D-16-001. The experiments were performed according to the guidelines of the Animal Care and Use Committee at KIOM.

### 4.4. Passive Cutaneous Anaphylaxis

The Ag/IgE-mediated PCA reaction was evaluated using a previous method [33]. ICR mice were subcutaneously injected through their ears with anti-DNP-IgE (100 ng) diluted in 1 × DPBS using an insulin syringe. On the next day, IgE-sensitized mice were orally administered KIOM-MA128 (0–100 mg/kg) or dexamethasone (10 mg/kg), and then intravenously administered 100 μg of DNP-HSA in 1 × DPBS containing 0.5% Evans blue 1 h later. After 30 min, the mice were euthanized by inhalation anesthesia; the ear was harvested and then incubated with 1 mL of formamide for 2 h at 80 °C. The mixture was homogenized and centrifuged for 10 min (17,000× *g*, 4 °C). The absorbance at 620 nm was measured using a SpectraMax i3 microplate reader (Molecular Devices, Sunnyvale, CA, USA).

### 4.5. Cell Culture

RBL-2H3 cells, a mast cell line originating from rat basophilic leukemia [34], were cultured in MEM-α medium containing 10% (*v*/*v*) FBS, 100 U/mL penicillin and 100 µg/mL streptomycin at 37 °C in a humidified atmosphere of 5% CO_2_. All the experiments contained a vehicle control group that was treated with 0.1% DMSO.

### 4.6. Cell Viability

Cell viability was determined by measuring the mitochondria-dependent reduction of WST-1 to the water-soluble tetrazolium salt [35]. Briefly, RBL-2H3 cells were seeded on a 96-well plate (1 × 10^4^ cells/well) in MEM-α medium with 10% FBS and incubated overnight at 37 °C. These cells were washed with 1× DPBS and then incubated with 1 μg/mL DNP-IgE for 24 h. The IgE-sensitized cells were preincubated with KIOM-MA128 (0–2000 μg/mL) in MEM-α medium containing 1% FBS for 1 h, and then simultaneously treated with 0.1 μg/mL DNP-HSA and 10 μL of the EZ-Cytox reagent and incubated for an additional 4 h. Cell viability was determined by measuring the absorbance at 450 nm using a microplate reader.

### 4.7. β-Hexosaminidase Activity

β-Hexosaminidase activity assay was evaluated using a previously reported method [36]. Supernatant (25 μL) was mixed with 50 μL of p-NAG (10 mM) in 0.1 M sodium citrate buffer (pH 4.5) in a 96-well plate and then incubated for 1 h at 37 °C. The reaction was terminated by the addition of stop buffer (0.1 M sodium carbonate buffer, pH 10.0). The absorbance was measured at 405 nm using a microplate reader.

### 4.8. Evaluation of Inflammatory Mediators

The IgE-sensitized cells were preincubated with KIOM-MA128 in MEM-α medium with 1% FBS for 1 h, spiked with DNP-HSA, and then incubated for an additional 4 h to determine the amounts of histamine, IL-4, IL-6, PGD_2_ or TNF-α in the conditioned media. All conditioned media were centrifuged (17,000× *g* at 4 °C) for 10 min, and the samples were stored at −80 °C until use. The histamine, IL-4, IL-6, PGD_2_ and TNF-α levels were detected using ELISA kits according to the manufacturer’s instructions.

### 4.9. Immunoblot Analysis

The immunoblot analysis was evaluated according to a previously described method [36]. The membranes were visualized by a chemiluminescent reaction (ECL plus kit, Bio-Rad, Hercules, CA, USA) and an imaging system (ChemiDoc Touch Imaging System, Bio-Rad). The levels of target proteins were compared to those of a loading control (β-actin), and the results were expressed as a ratio of the density of each protein identified using a protein standard size marker (BIOFACT, Daejeon, Korea). The density of each band was measured using ImageJ software (version 1.49v for Windows, National Institutes of Health (NIH), Bethesda, MD, USA).

### 4.10. Statistical Analysis

All the experimental results are listed as the mean ± SD or SEM. One-way analysis of variance (ANOVA) was used for multiple comparisons (GraphPad Prism version 5.03 for Windows, GraphPad Software Inc., San Diego, CA, USA). The Dunnett test was applied when there was a significant variation between the treated groups.

## 5. Conclusions

The present study shows that KIOM-MA128 exerts its anti-allergic properties by inhibiting both the FcεRI and arachidonate cascades in Ag/IgE-activated mast cells in vitro and in vivo. These findings provide the clue regarding the anti-allergic actions of KIOM-MA128 in asthma and atopic dermatitis. The anti-allergic mechanisms in Ag/IgE-activated mast cells may include several targets, such as LYN, FYN, SYK, MAPKs, cPLA2 and COX-2. Moreover, the anti-allergic effects of KIOM-MA128 may result from the combination of the herbal components, including the anti-allergic actions produced by the fermentation process. Therefore, further study is necessary to reveal the major component of KIOM-MA128 that is responsible for its anti-allergic actions.
